# Prevalence of Sleep Disturbance and Associated Risk Factors in Degenerative Cervical Myelopathy

**DOI:** 10.3390/jcm14197110

**Published:** 2025-10-09

**Authors:** Salim Yakdan, Karan Joseph, Jingyi Zhang, Miguel A. Ruiz-Cardozo, Aryan Pradhan, Alisha Dhallan, Faraz Arkam, William Mualem, Garrison Bentz, Diogo P. Moniz Garcia, Benjamin Plog, Alexander T. Yahanda, Daniel Hafez, Wilson Z. Ray, Camilo A. Molina, Jacob K. Greenberg

**Affiliations:** Department of Neurosurgery, Washington University School of Medicine, St. Louis, MO 63110, USA

**Keywords:** cervical meylopathy, sleep, migraine

## Abstract

Study Design: Retrospective Case–Control. **Objectives:** Sleep disturbances negatively impact quality of life and increase illness susceptibility. Chronic pain is a risk factor for sleep disruption, particularly in patients with degenerative spinal conditions. Existing studies suggest that degenerative cervical myelopathy (DCM) patients often experience sleep disturbances, possibly due to spinal cord compression and pain. However, most research is limited to small, single-center studies, creating a need for broader analyses. **Methods:** We utilized the Merative Explorys Dataset, focusing on electronic health record data of patients diagnosed with DCM and sleep disorders identified via ICD codes. Comorbidities analyzed included depression/bipolar disorder, chronic pulmonary disease, migraine, osteoarthritis, hypertension, malignancy, diabetes, and cerebrovascular disease. Patient demographic information (age, race, sex, and body mass index (BMI)) was included as covariates. Logistic regression analyses were performed to evaluate the association between each comorbidity and the risk of sleep disturbance. **Results:** Among 40,551 DCM patients, significant predictors of sleep disturbance included higher BMI (OR: 1.05, 95% CI: 1.05–1.06), depression/bipolar disorder (OR: 1.65, 95% CI: 1.56–1.74), chronic pulmonary disease (OR: 1.26, 95% CI: 1.20–1.33), migraine (OR: 1.32, 95% CI: 1.22–1.43), and hypertension (OR: 1.16, 95% CI: 1.10–1.23). **Conclusions:** This large-scale analysis demonstrates the multifactorial nature of sleep disturbances in DCM, highlighting strong associations with BMI and respiratory conditions, suggesting a contributory role of sleep-disordered breathing. The identification of migraines as a risk factor highlights the need for multidisciplinary management. Addressing modifiable risk factors such as BMI and mental health may improve sleep quality in DCM patients.

## 1. Introduction

Sleep is an essential function of the human body and plays a major role in maintaining cognitive function and physiologic processes such as glucose metabolism and overall metabolic regulation [1,2]. Given its role in multiple physiological domains, sleep disturbance and deficits are associated with decreased quality of life, increased illness susceptibility, and increased mortality [3,4,5]. Sleep deprivation is a global issue affecting nearly 20% of people across nations [6]. In the United States, only 65.2% of individuals report healthy sleep, and more than one-third of respondents report sleeping less than seven hours per night [6,7].

Among the numerous established risk factors for sleep disturbance, chronic pain is of particular interest. Approximately 50% of patients with chronic low back pain experience significant sleep disturbance, while 42% of individuals with chronic neck pain and chronic back pain report similar problems. Similarly, studies have demonstrated that sleep disturbances are prevalent in patients with degenerative spinal disease, including degenerative cervical myelopathy (DCM), lumbar spinal stenosis, and adult spinal deformity [8,9,10]. These studies propose that the mechanisms driving sleep disturbances in DCM patients may be related to pain, paresthesias, sleep breathing disorders and sleep movement disorders, or the possible disruption of melatonin pathways in the cervical spinal cord. Another hypothesis is that cervical stenosis may alter cerebrospinal fluid dynamics, impairing waste clearance and increasing posterior fossa pressure, which could in turn exacerbate sleep apnea and other sleep-related disturbances [11]. Along these lines, surgically treated patients who receive decompression of the spinal cord show greater improvements in sleep disturbance symptoms than those managed conservatively [12].

Sleep disturbances have long been overlooked in spine surgery literature, where most of the studies focus on surgical outcomes [13,14,15,16,17,18,19,20,21]. Available data remain inadequate, especially regarding DCM. The majority of studies on the cervical spine examine cervical spinal cord injury [22,23,24]. When focusing specifically on DCM, what sparse literature exists is restricted by single-centered studies with small patients samples [25], which may introduce bias in epidemiological analyses and risk factor identification. Accordingly, augment this literature, our study sought to leverage a large population-based patient dataset to identify risk factors of sleep disturbance in patients with DCM.

## 2. Materials and Methods

### 2.1. Study Design and Population

This retrospective case–control study leveraged the Merative Explorys Therapeutic Dataset, a large de-identified, population-based repository aggregating electronic health records (EHRs) from multiple U.S. healthcare systems. Adult patients with a diagnosis of DCM were identified using International Classification of Diseases, Tenth Revision (ICD-10) codes. In order to reduce the likelihood of including sleep disorders that may be unrelated to underlying DCM, relevant cases were defined as individuals who received a subsequent diagnosis of a sleep disorder (also identified via ICD-10 codes) at least six months after the index date of DCM diagnosis. Controls were selected from the same DCM cohort and included patients who did not receive any sleep disorder diagnosis after being diagnosed with DCM. Demographic and comorbidities were extracted from EHR. Supplementary Table S1 provides the ICD-10 codes used.

### 2.2. Statistical Analysis

All statistical analyses were performed using reproducible scripts in R Studio (v4.2 2024.02.29) First, descriptive analyses were performed to characterize the demographic and clinical profiles of the study population. Continuous variables were summarized using means and standard deviations (SD) or medians and interquartile ranges (IQR) as appropriate, while categorical variables were described using frequencies and percentages. Univariable analyses were performed to explore the association between each potential risk factor and the likelihood of developing sleep disorders following a diagnosis of DCM.

For multivariable analyses, a logistic regression model was constructed to estimate the adjusted associations between candidate predictors and the risk of sleep disorders among DCM patients. The independent variables used in the model were those considered clinically relevant or consistently reported in the literature. To ensure model stability and interpretability, two variable selection methods were employed. First, a stepwise selection approach was used to iteratively refine the model based on the Akaike Information Criterion (AIC). Collinearity diagnostics, including variance inflation factors (VIFs), were examined to ensure that no excessive correlation existed among the predictors. Final estimates were reported as adjusted odds ratios (OR) with 95% confidence intervals (95% CI). Statistical significance was defined as *p* < 0.05.

### 2.3. Missing Data Handling

To address missing values, a multiple imputation strategy was employed. Ten imputed datasets were created using a chained equations approach, wherein each incomplete variable was regressed on other variables iteratively. Each dataset underwent 50 iterations to refine the imputed values, thereby capturing uncertainty and improving accuracy. After imputation, the 10 completed datasets were combined using Rubin’s rules to produce final estimates.

## 3. Results

### 3.1. Descriptive Characteristics of the Study Population

**Figure 1:**
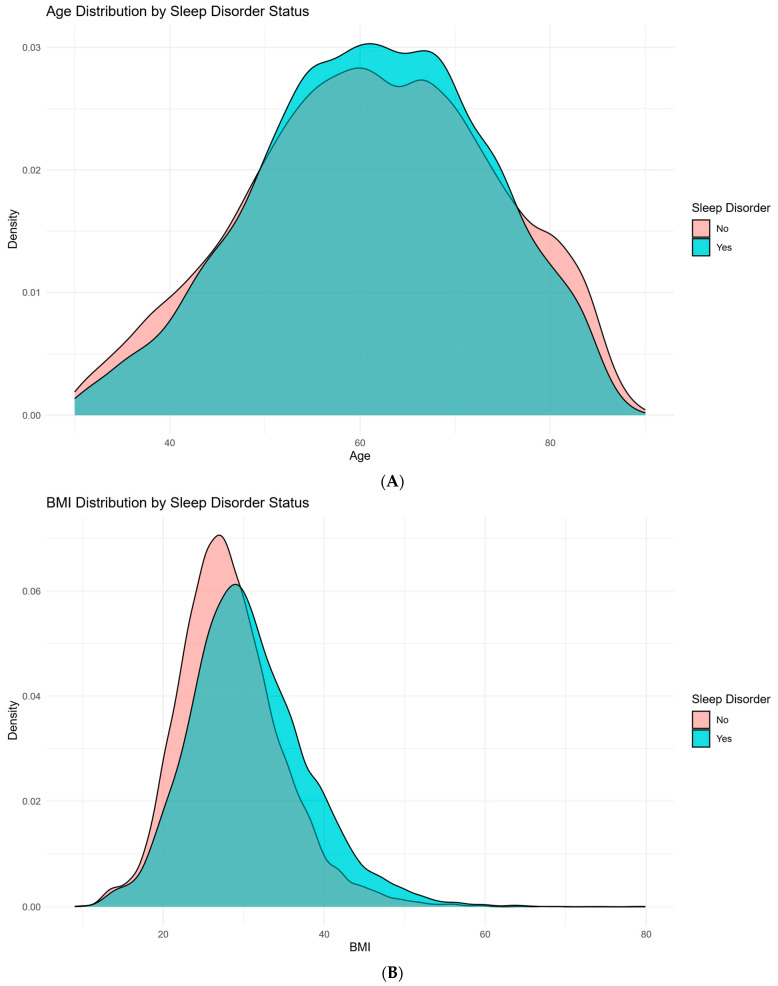

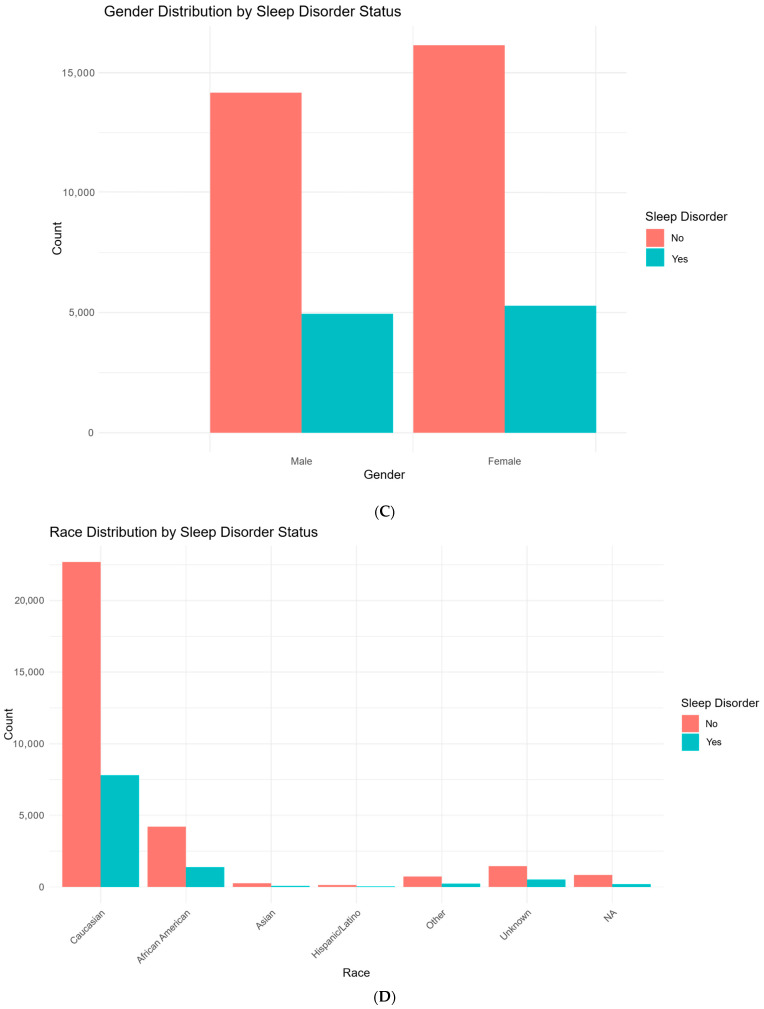

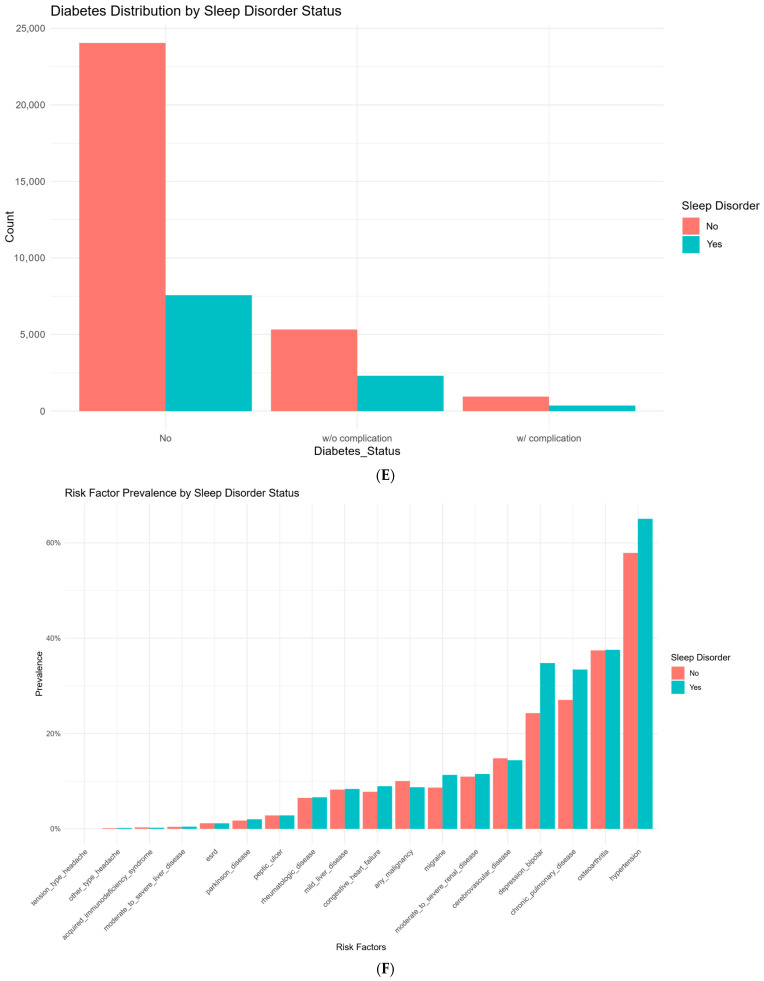
Demographic and clinical characteristics stratified by sleep disorder status in patients with degenerative cervical myelopathy (DCM). (**A**): Distribution of age in DCM patients, comparing those with and without sleep disorders. (**B**): Distribution of body mass index (BMI) in DCM patients, comparing those with and without sleep disorders. (**C**): Distribution of gender in DCM patients, comparing those with and without sleep disorders. (**D**): Distribution of race in DCM patients, comparing those with and without sleep disorders. (**E**): Prevalence of Diabetes Status (no diabetes, diabetes without complications, and diabetes with complications) in DCM patients with and without sleep disorders. (**F**): Distribution of risk factor prevalence in DCM patients, comparing those without and without sleep disorders.

A total of 40,551 patients with DCM were included in this study. Table 1 and Figure 1 summarize the demographic and clinical characteristics of the study population stratified by the presence or absence of sleep disorders following DCM diagnosis. Of the total cohort, 74.8% (n = 30,322) did not develop sleep disorders, while 25.2% (n = 10,229) did. The median (IQR) age was 61 (53–70) years in the group with sleep disorders was 61 (52–71) years in the group without. Body mass index (BMI) was significantly higher among patients who developed sleep disorders, with a median (IQR) of 30.0 (25.8–35.1), compared to those without sleep disorders, whose median BMI was 27.8 (24.1–32.0) (*p* < 0.001).

Several comorbidities were more prevalent in the sleep disorder group. Hypertension was present in 65.1% of those with sleep disorders, compared to 57.9% in the non-sleep disorder group (*p* < 0.001). Likewise, conditions such as congestive heart failure (*p* < 0.001), chronic pulmonary disease (*p* < 0.001), and migraine (*p* < 0.001) were more common in individuals who developed sleep disorders. Notably, depression or bipolar disorder was reported in 34.8% of patients with sleep disorders compared to 24.3% of those without (*p* < 0.001).

### 3.2. Univariable Analysis

**Figure 2:**
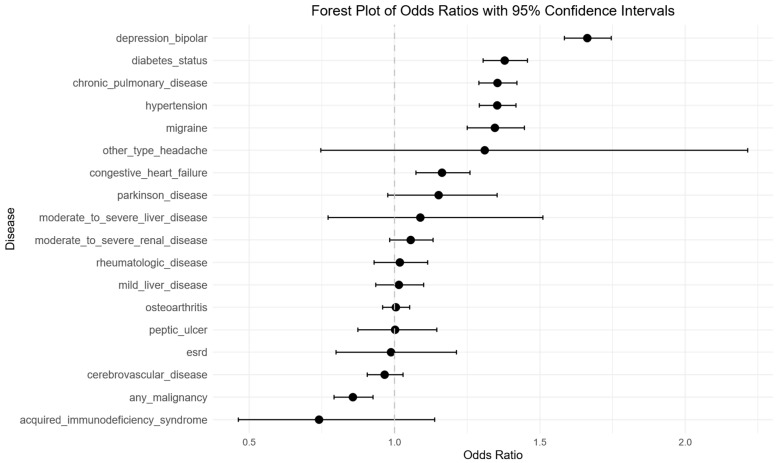
Univariable logistic regression analysis of risk factors for sleep disorder in degenerative cervical myelopathy patients. Forest plot illustrating odds ratios (OR’s) and 95% confidence intervals (CIs) for each evaluated predictor, demonstrating statistically significant associations with depression/bipolar, diabetes status, chronic pulmonary disease, hypertension, migraine. Negative associations were seen with any malignancy.

Table 2 and Figure 2 present the OR and 95% CI for each predictor evaluated in the univariable logistic regression analysis. Depression or bipolar disorder (OR: 1.66, 95% CI: 1.58–1.75; *p* < 0.001) and diabetes (OR: 1.38, 95% CI: 1.30–1.46; *p* < 0.001) were strongly associated with sleep disorders. Chronic pulmonary disease (OR: 1.35, 95% CI: 1.29–1.42; *p* < 0.001) and hypertension (OR: 1.35, 95% CI: 1.29–1.42; *p* < 0.001) were each associated with elevated odds of sleep disorders, as was migraine (OR: 1.35, 95% CI 1.25–1.45; *p* < 0.001). Body mass index (BMI) demonstrated a positive association, with each unit increase linked to a higher risk of sleep disorders (OR 1.05, 95% CI: 1.05–1.05; *p* < 0.001). Congestive heart failure also showed a modest but statistically significant association (OR 1.16, 95% CI: 1.07–1.26; *p* < 0.001).

### 3.3. Multivariable Analysis

**Figure 3:**
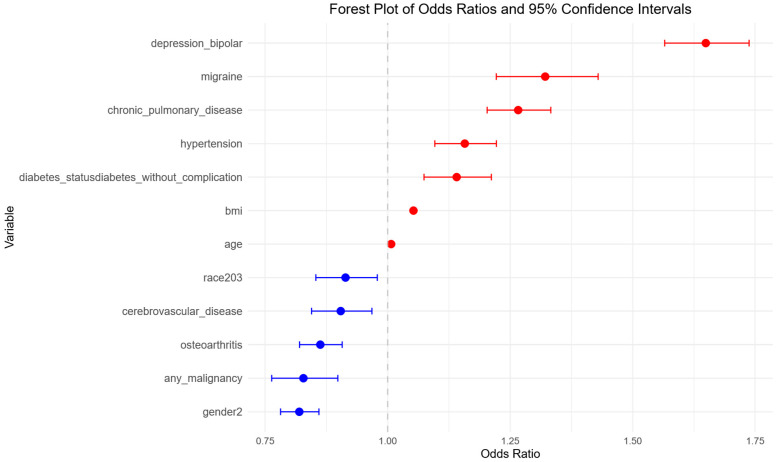
Multivariable logistic regression analysis of risk factors for sleep disorders in degenerative cervical myelopathy patients. Forest plot showing adjusted odds ratios (ORs) and 95% confidence intervals (CIs) from the final logistic regression model, highlighting independent predictors of sleep disorders. Blue points represent predictors with ORs < 1.00. Red points represent predictors with ORs ≥ 1.00.

A multivariable logistic regression model was constructed using predictors selected through a stepwise procedure. This yielded a final model that identified several significant sleep disorder predictors (Figure 3). Among these, chronic pulmonary disease (OR: 1.27, 95% CI: 1.20–1.33, *p* < 0.001), depression or bipolar disorder (OR: 1.65, 95% CI: 1.57–1.74, *p* < 0.001), migraine (OR: 1.32, 95% CI: 1.22–1.43, *p* < 0.001), hypertension (OR: 1.16, 95% CI: 1.10–1.22, *p* < 0.001), and diabetes without complications (OR: 1.14, 95% CI: 1.07–1.21, *p* < 0.001) were associated with an increased likelihood of developing sleep disorders. Higher BMI and older age were also positively related to risk (OR of approximately 1.05 per unit increase in BMI and 1.01 per year increase in age) (*p* < 0.001 for both).

In contrast, osteoarthritis (OR: 0.86, 95% CI: 0.82–0.91, *p* < 0.001), any malignancy (OR: 0.83, 95% CI: 0.76–0.90, *p* < 0.001), cerebrovascular disease (OR: 0.90, 95% CI: 0.84–0.97, *p* = 0.004), and being African American compared to Caucasian (OR: 0.91, 95% CI: 0.85–0.98, *p* = 0.01), and female gender (OR: 0.82, 95% CI: 0.78–0.86, *p* < 0.001) were associated with reduced risk of sleep disorder.

## 4. Discussion

To our knowledge, this study provides the largest analysis examining risk factors associated with sleep disturbance in patients with DCM. Using a robust dataset of 40,551 DCM patients, we identified several significant predictors of sleep disturbance, including higher BMI, depression or bipolar disorder, chronic pulmonary disease, migraines, and hypertension. These findings expand our understanding of the multifactorial nature of sleep disorders in DCM patients, underscoring the role that both physiologic and psychological comorbidities play in sleep outcomes.

Our logistic regression analysis revealed that higher BMI (OR: 1.05, 95% CI: 1.05–1.06), depression or bipolar scores (OR: 1.65, 95% CI: 1.56–1.74), chronic pulmonary disease (OR 1.26 95% CI: 1.20–1.33), migraine (OR 1.32, 95% CI 1.22–1.43), and hypertension (OR 1.16, 95% CI 1.10–1.23) were independently associated with increased odds of sleep disturbance. These risk factors align with findings from prior research on degenerative spinal disease, but our study further highlights their relevance to patients suffering specifically from DCM [9,26]. Additionally, we found that age, although statistically significant, had only a modest effect size (OR: 1.01, 95% CI 1.00-1.01) suggesting that its clinical impact may be less profound than reported in other studies [27].

The mechanisms underlying sleep disturbance in DCM are likely complex and multifactorial, including both neurological and systemic factors. Emerging evidence highlights the role of microglial regulation and neuroinflammation in sleep disturbance across neurological conditions. Microglia influence sleep–wake regulation and become activated in response to stress and sleep fragmentation or deprivation. This activation can lead to release of pro-inflammatory cytokines and further disrupt sleep [28,29,30]. In the context of DCM, chronic spinal cord compression may similarly engage these pathways, suggesting that neuroinflammatory cascades could represent an additional biological mechanism linking DCM to sleep disturbance.

We complement this mechanistic discussion by identifying key risk factors for sleep disturbance in this population. Our results are consistent with the findings of Kim et al., who identified depression, chronic pain, and neuropsychiatric conditions to be significant predictors of sleep disturbance in patients with spinal disease [27]. Our study builds on these results by incorporating other risk factors such as chronic pulmonary disease and hypertension, which have not been emphasized in previous studies on sleep disturbances in DCM patients. The significant associations between sleep disturbance and chronic pulmonary disease and BMI suggest that respiratory conditions, potentially through mechanisms such as sleep-disordered breathing, hypersomnias, or obesity hypoventilation syndrome, play a notable role in exacerbating sleep issues in this population [31,32].

Furthermore, our findings regarding migraines are novel in this population and align with broader research which have found migraines to have a major impact on sleep [33,34]. Altered sleep architecture, pain associated with migraines [35], and disruptions in calcitonin gene-related peptide (CGRP) likely contribute to poor sleep quality [35,36]. Prior studies focusing on spinal pathology have not substantially published on this link, making our study an additional notable iteration upon prior work.

Given the growing recognition of mental health as a critical determinant of outcomes in spine surgery populations [37,38,39,40], our findings emphasize a significant association between depression or bipolar disorder and sleep disturbances in DCM. Depression is well-documented to disrupt sleep architecture, contributing to difficulties with both sleep initiation and maintenance. In the context of DCM, this psychological burden may be further amplified by chronic pain and neurological symptoms, compounding the risk for sleep disruption. These results underscore the need for routine mental health screening and management in this population [41].

The identification of these risk factors has clinical implications for the management of DCM patients. Addressing modifiable risk factors such as BMI should be a priority in the pre- and perioperative period. Managing patient’s weight could potentially reduce the risk of sleep related breathing disorders and improve sleep quality. As in spinal cord injury, DCM may result in neural pathway disruption which may impair circadian regulation through altered melatonin production [42,43]. Pain and paresthesias, which are hallmarks of DCM, contribute to sleep disturbances by increasing nocturnal arousals and reducing sleep quality.

Given the associations between these multiple different conditions (some of which tend to occur together in a comorbid fashion) and sleep disturbance in DCM, a multidisciplinary approach is necessary to address this problem. Collaborations across multiple specialties could address the broad spectrum of comorbidities that contribute to sleep disturbance in this patient population. Screening these patients beforehand and connecting them with the right provider to preemptively treat these underlying comorbidities could significantly enhance patient quality of life. In practice, the identified predictors may serve as a framework for risk stratification: for example, patients with elevated BMI or chronic pulmonary disease could be prioritized for evaluation of sleep-disordered breathing, while those with depression or migraines may benefit from early referral to psychiatry or neurology. This could improve early recognition, allow proactive intervention, and ensure that high-risk patients are stratified appropriately for perioperative management.

Interestingly, certain conditions such as osteoarthritis (OR: 0.86, 95% CI: 0.82–0.91), any malignancy (OR: 0.83, 95% CI: 0.76–0.90), cerebrovascular disease (OR: 0.90, 95% CI: 0.84–0.97), and Black race compared to White (OR: 0.91, 95% CI: 0.85–0.97) were associated with lower odds of sleep disturbance. While these findings may initially appear counterintuitive, it is possible that patients with these conditions are more likely to be prescribed medications such as opioids, muscle relaxants, or sedating agents, which may incidentally improve sleep symptoms. Alternatively, these associations may reflect unmeasured confounders or biases in reporting. Future studies should consider integrating medication profiles and longitudinal follow-up to better understand these inverse relationships.

### Limitations

Our study has several limitations. The retrospective nature of the data collection introduces potential bias, particularly in the accuracy of recorded diagnoses. Additionally, we were unable to include objective measures of sleep quality such as polysomnography, which would have provided a more direct assessment of sleep disturbances in this population. Further research with prospective data and objective sleep assessments is needed to confirm the results of our study. Moreover, we were unable to account for medication use in this cohort, including antidepressants, hypnotics, and analgesics, which may be important confounding factors influencing sleep. Another important limitation is that our study did not examine the severity of DCM with sleep disturbance. This is relevant, as one might expect patients with more severe disease to have worse sleep outcomes. However, even with patients being categorized as having severe DCM, assessment can be challenging [44]. Future studies incorporating validated severity scales alongside objective sleep measures should be conducted. Another limitation is our reliance on ICD coding to identify comorbidities such as depression or migraine. This may introduce bias, with the potential for both under- and overestimation of these conditions; however, it is an acceptable and widely used method in large population studies [45,46,47,48]. Lastly, interventions aimed at modulating the identified risk factors, particularly in patients undergoing surgical intervention for DCM, should be evaluated for their impact on sleep outcomes.

## 5. Future Directions and Applications

Future work should move behind large sized retrospective HER analyses and expand to prospective cohort studies that integrate both clinical and biological markers of sleep disturbance in DCM. This could include objective sleep assessments, serum or CSF biomarkers of neuroinflammation, and validated scales of DCM severity (Modified Japanese Orthopedic Association Score) to better capture the link between disease burden and sleep outcomes. In parallel, translational studies examining the role of microglial activation, circadian disruption and cerebrospinal fluid dynamics may provide mechanistic insight into the pathways underlying sleep disturbance in this population.

Clinically, our findings highlight opportunities for earlier intervention. Risk factors such as BMI, pulmonary disease and depression could be incorporated into preoperative screening tools to identify patients at greatest risk for poor sleep quality. These predictors may also be used to stratify patients and tailor perioperative management; for example, through targeted weight optimization, pulmonary evaluation, and depression or migraine management via specialty care. Such strategies may ultimately improve not only sleep quality but also overall recovery trajectories and quality of life for patients with DCM.

## 6. Conclusions

Our study utilizes a large dataset of patients with DCM to provide robust evidence that BMI, depression/bipolar status, chronic pulmonary disease, migraines, and hypertension are significant predictors of sleep disturbance for these patients. Our findings highlight the multifactorial nature of sleep disorders in this population and underscore the need for multidisciplinary approaches to the pre- and perioperative care of DCM patients as well as further research regarding DCM and sleep.

## Figures and Tables

**Table 1:** Descriptive Statistics of Potential Risk Factors for Sleep Disturbance in Degenerative Cervical Myelopathy patients. Demographic details, clinical characteristics, and comorbidity prevalence stratified by the presence or absence of subsequent sleep disorders.

	Level (Code)	Without Sleep Disorder	With Sleep Disorder	*p* Value
n	-	30,322 (74.77)	10,229 (25.23)	
Age (median (Q1, Q3))	-	61 (52, 71)	61 (53, 70)	0.9113
Age (mean (SD))	-	61.14 (12.79)	61.18 (11.88)	
BMI (median (Q1, Q3))	-	27.79 (24.12, 32.03)	30 (25.82, 35.08)	6.49 × 10^−179^
BMI (mean (SD))	-	28.43 (6.45)	30.78 (7.40)	
Hemiplegia Paraplegia (%)	-	313 (1.03)	75 (0.73)	0.0086
Hypertension (%)	-	17,557 (57.90)	6654 (65.05)	3.80 × 10^−37^
Myocardial Infarction (%)	-	2040 (6.73)	706 (6.90)	0.5596
Congestive Heart Failure (%)	-	2361(7.79)	915 (8.95)	0.0002
Peripheral Vascular Disease (%)	-	3521 (11.61)	1087 (10.63))	0.0070
Cerebrovascular Disease (%)	-	4494 (14.82)	1472 (14.39)	0.2953
Dementia (%)	-	1060 (3.5)	296 (2.89)	0.0038
Chronic Pulmonary Disease (%)	-	8196 (27.03)	3417 (33.41)	7.01 × 10^−35^
Rheumatologic Disease (%)	-	1970 (6.50)	676 (6.61)	0.7095
Peptic Ulcer (%)	-	855 (2.82)	289 (2.83)	1
Mild Liver Disease (%)	-	2500 (8.24)	855 (8.36)	0.7336
Moderate to Severe Liver Disease (%)	-	128 (0.42)	47 (0.46)	0.6811
Moderate to Severe Renal Disease (%)	-	3325 (10.97)	1177 (11.51)	0.1369
ESRD (%)	-	357 (1.18)	119 (1.16)	0.9517
Any Malignancy (%)	-	3045 (10.04)	893 (8.73)	0.0001
Metastatic Solid Tumor (%)	-	667 (2.20)	153 (1.50)	1.47 × 10^−5^
Acquired Immunodeficiency Syndrome (%)	-	96 (0.32)	24 (0.23)	0.224514637
Parkinson Disease (%)	-	529 (1.74)	205 (2.00)	0.0970
Migraine (%)	-	2628 (8.67)	1158 (11.32)	1.75 × 10^−15^
Tension Type Headache (%)	-	0 (0)	0 (0)	
Other Type Headache (%)	-	43 (0.14))	19 (0.19)	0.4025
Osteoarthritis (%)	-	11,350 (37.43)	3840 (37.54)	0.8535
Depression Bipolar (%)	-	7358 (24.27)	3556 (34.76)	4.48 × 10^−95^
Race (%)	Caucasian	22,695 (74.85)	7801 (76.26)	0.1580
	African American	4209 (13.88)	1382 (13.51)	
	Asian	260 (0.86)	72 (0.70)	
	Hispanic/Latino	135 (0.45)	36 (0.35)	
	Other	731 (2.41)	228 (2.23)	
	Unknown	1453 (4.79)	515 (5.03)	
	NA	839 (2.77)	195 (1.91)	
Gender (%)	Unknown	3 (0.01)	0 (0.0)	0.0109
	Male	14,170 (46.73)	4946 (48.35)	
	Female	16,149 (53.26)	5283 (51.65)	
Diabetes Status (%)	No Diabetes	24051 (79.3)	7562 (73.9)	8.15 × 10^−30^
	Diabetes Without Complication	5323 (17.6)	2308 (22.6)	
	Diabetes With Complication	948 (3.1)	359 (3.5)	

**Table 2:** Univariable Logistic Regression Results of Predictors of Sleep Disturbance in Degenerative Cervical Myelopathy patients: Provides coefficients, odds ratios (ORs), 95% confidence intervals (CIs), and *p*-values for each variable tested for association with sleep disorder.

Variable	Coefficient	*p* Value	Odds Ratio Confidence Interval
Hypertension	0.303	0	1.35 (1.29–1.42)
Chronic Pulmonary Disease	0.303	0	1.35 (1.29–1.42)
Migraine	0.297	0	1.35 (1.25–1.45)
Depression Bipolar	0.509	0	1.66 (1.58–1.75)
BMI	0.05	0	1.05 (1.05–1.05)
Diabetes Status	0.321	0	1.38 (1.30–1.46)
Any Malignancy	−0.155	1.00 × 10^−4^	0.86 (0.79–0.93)
Congestive Heart Failure	0.151	2.00 × 10^−4^	1.16 (1.07–1.26)
Parkinson Disease	0.141	0.0889	1.15 (0.98–1.36)
Moderate to Severe Renal Disease	0.054	0.1322	1.06 (0.98–1.13)
Acquired Immunodeficiency Syndrome	−0.3	0.1885	0.74 (0.47–1.16)
Race	−0.042	0.2103	0.96 (0.90–1.02)
Cerebrovascular Disease	−0.035	0.2879	0.97 (0.91–1.03)
Other Type Headache	0.27	0.3269	1.31 (0.76–2.25)
Moderate to Severe Liver Disease	0.085	0.6184	1.09 (0.78–1.52)
Rheumatologic Disease	0.018	0.6924	1.02 (0.93–1.11)
Mild Liver Disease	0.015	0.718	1.02 (0.94–1.10)
Age	0	0.7339	1.00 (1.00–1.00)
Osteoarthritis	0.005	0.8442	1.00 (0.96–1.05)
Gender	−9.513	0.8903	0.00 (0.00–3.81 × 10^54^)
ESRD	−0.012	0.9095	0.99 (0.80–1.22)
Peptic Ulcer	0.002	0.9765	1.00 (0.88–1.15)

## Data Availability

The data used in this study were obtained from the Merative™ MarketScan^®^ Research Databases and are available to licensed users through commercial agreement with Merative.

## Supplementary Materials

The following supporting information can be downloaded at: https://www.mdpi.com/article/10.3390/jcm14197110/s1, Table S1: ICD-10 Codes of Comorbid Conditions: ICD-10 codes of comorbid conditions. Comprehensive list of ICD-10 codes used to identify each comorbid conditions included in this study.
