# Supplementary material for: Prevalence of Sleep Disturbance and Associated Risk Factors in Degenerative Cervical Myelopathy
Source: J Clin Med. 2025 Oct 9;14(19):7110. doi: 10.3390/jcm14197110 (PMC12525738; doi:10.3390/jcm14197110)
Supplement: Supplementary file 1 [file jcm-14-07110-s001.zip › jcm-3883162-supplementary.pdf]

Supplementary Table S1: ICD-10 Codes of Comorbid Conditions: ICD-10 codes of comorbid conditions. Comprehensive list of ICD-10 codes used to identify each comorbid conditions included in this study.	
**Comorbid Conditions**	**ICD-10 Codes**
Sleep Disorder	F51%, G47%
Diabetes without Complication	E12, E12.9, E13, E13.9, E10, E10.9, E11, E11.9, E14, E14.9
Diabetes with complication	M14.2, H28.0, G59.0, G63.2, H36.0, N08.3, E12.0, E12.1, E12.7, E12.4, E12.3, E12.6, E12.5, E12.2, E12.8, E13.0,<br>E13.1, E13.7, E13.4, E13.3, E13.6, E13.5, E13.2, E13.8, E10.0, E10.1, E10.7, E10.4, E10.3, E10.6, E10.5, E10.2, E10.8,<br>E11.0, E11.1, E11.7, E11.4, E11.3, E11.6, E11.5, E11.2, E11.8, E14.0, E14.1, E14.7, E14.4, E14.3, E14.6, E14.5, E14.2,<br>E14.8
Hypertension	I10, I11, I11.0, I11.9, I12, I12.9, I12.0, I13, I13.9, I13.2, I13.0, I13.1, I15.2, I15.1, I15.8, I15.0, I15, I15.9
Congestive Heart Failure	I13.0, I13.2, I11.0, I25.5, I42.0, I42.5, I42.6, I42.7, I42.8, I42.9, I43, I43.0, I43.1, I43.2, I43.8, I50, I50.0, I50.9, I50.1,<br>P29.0
Cerebrovascular Disease	G45%, G46%, I60%, I61%, I62%, I63%, I64%, I65%, I66%, I67%, I68%, I69%
Chronic Pulmonary Disease	J40%, J41%, J42%, J43%, J44%, J45%, J46%, J47%, J60%, J61%, J62%, J63%, J64%, J65%, J66%, J67%, J68.4%,<br>J70.1%, J70.3%

Rheumatologic Disease	M05%, M06%, M31.5%, M32%, M33%, M34%, M35.1%, M35.3%
Peptic Ulcer	K25%, K26%, K27%, K28%
Mild Liver Disease	B18%, K70.0%, K70.1%, K70.2%, K70.3%, K70.9%, K71.3%, K71.4%, K71.5%, K71.7%, K73%, K74%, K76.0%, K76.2%, K76.3%, K76.4%, K76.8%, K76.9%, Z94.4%
Moderate to Severe Liver Disease	I85.0, I85.9, I85, I86.4, I98.2, K70.4, K71.1, K72.1, K72.9, K76.5, K76.6, K76.7
Moderate to Severe Renal Disease	I12.0, I13.1, I13.2, N03.2, N03.3, N03.4, N03.5, N03.6, N03.7, N05.2, N05.3, N05.4, N05.5, N05.6, N05.7, N05.8, N05.9, N18, N18.1, N18.2, N18.3, N18.4, N18.5, N18.9, N18.0, N18.8, N19, N25.0, Z49, Z49.2, Z49.0, Z49.1, Z94.0, Z99.2
ESRD (End-Stage Renal Disease)	N18.5, N18.0, Z49, Z49.2, Z49.0, Z49.1, Z99.2
Any Malignancy	C00%, C01%, C02%, C03%, C04%, C05%, C06%, C07%, C08%, C09%, C10%, C11%, C12%, C13%, C14%, C15%, C16%, C17%, C18%, C19%, C20%, C21%, C22%, C23%, C24%, C25%, C26%, C30%, C31%, C32%, C33%, C34%, C37%, C38%, C39%, C40%, C41%, C43%, C45%, C46%, C47%, C48%, C49%, C50%, C51%, C52%, C53%, C54%, C55%, C56%, C57%, C58%, C60%, C61%, C62%, C63%, C64%, C65%, C66%, C67%, C68%, C69%, C70%, C71%, C72%, C73%, C74%, C75%, C76%, C81%, C82%, C83%, C84%, C85%, C88%, C90%, C91%, C92%, C93%, C94%, C95%, C96%, C97%

Acquired Immunodeficiency Syndrome	B20%, B21%, B22%, B24%
Parkinson Disease	G20, G21.8, G22, G21, G21.2, G21.9
Migraine	G43%
Tension-Type Headache	G44.2
Other Type Headache	G44.0, G44.1, G44.3, G44.4, G44.8
Osteoarthritis	M15%, M16%, M17%, M18%, M19%
Depression/Bipolar	F31.3, F31.4, F31.5, F92.0, F32, F32.9, F32.0, F41.2, F32.1, F32.8, F33.8, F20.4, F33, F33.0, F33.1, F33.2, F33.3, F33.4,<br>F33.9, F25.1, F32.2, F32.3, F31, F31.0, F31.1, F31.2, F31.6, F31.9, F31.8
